# Chemotherapy-Induced Late Transgenerational Effects in Mice

**DOI:** 10.1371/journal.pone.0017877

**Published:** 2011-03-17

**Authors:** Loro L. Kujjo, Eun A. Chang, Ricardo J. G. Pereira, Shilpa Dhar, Brenda Marrero-Rosado, Satyaki Sengupta, Hongbing Wang, Jose B. Cibelli, Gloria I. Perez

**Affiliations:** 1 Department of Physiology, Michigan State University, East Lansing, Michigan, United States of America; 2 Department of Human Anatomy, Michigan State University, East Lansing, Michigan, United States of America; 3 Department of Animal Sciences, Michigan State University, East Lansing, Michigan, United States of America; 4 LARCel, Programa Andaluz de Terapia Celular y Medicina Regenerativa, Sevilla, Spain; University of Windsor, Canada

## Abstract

To our knowledge, there is no report on long-term reproductive and developmental side effects in the offspring of mothers treated with a widely used chemotherapeutic drug such as doxorubicin (DXR), and neither is there information on transmission of any detrimental effects to several filial generations. Therefore, the purpose of the present paper was to examine the long-term effects of a single intraperitoneal injection of DXR on the reproductive and behavioral performance of adult female mice and their progeny. C57BL/6 female mice (generation zero; G0) were treated with either a single intraperitoneal injection of DXR (G0-DXR) or saline (G0-CON). Data were collected on multiple reproductive parameters and behavioral analysis for anxiety, despair and depression. In addition, the reproductive capacity and health of the subsequent six generations were evaluated. G0-DXR females developed despair-like behaviors; delivery complications; decreased primordial follicle pool; and early lost of reproductive capacity. Surprisingly, the DXR-induced effects in oocytes were transmitted transgenerationally; the most striking effects being observed in G4 and G6, constituting: increased rates of neonatal death; physical malformations; chromosomal abnormalities (particularly deletions on chromosome 10); and death of mothers due to delivery complications. None of these effects were seen in control females of the same generations. Long-term effects of DXR in female mice and their offspring can be attributed to genetic alterations or cell-killing events in oocytes or, presumably, to toxicosis in non-ovarian tissues. Results from the rodent model emphasize the need for retrospective and long-term prospective studies of survivors of cancer treatment and their offspring.

## Introduction

Chemicals used in the treatment of cancer are unquestionably beneficial as therapeutic agents. Nevertheless, the ensuing detrimental reproductive and developmental problems for both the treated mothers and their offspring cannot be ignored [1], [2], . In the USA it is estimated that by the end of 2010, 1-in-60 women under the age of 39 will be a cancer survivor who has been exposed to a chemotherapeutic agent [14], [15]. How many of those survivors of cancer treatment will become pregnant remains unknown; however, it is grossly estimated that at least half of them will do so [16], [17], [18]. Unfortunately, there is a paucity of information on the effects of chemotherapeutic agents on the offspring of cancer survivors that received chemotherapy.

Therefore, the present experiments were designed to examine the long-term effects of a single intraperitoneal injection of DXR on the reproductive and behavioral performance of adult female mice and their progeny. We observed that when young adult females are treated with doxorubicin (DXR), a widely used chemotherapeutic agent [19], the animals develop dysfunctions in multiple organs, including the brain, uterus, and ovaries. Surprisingly, the doxorubicin-induced effects in oocytes were transmitted transgenerationally by both males and females born to a chemo-treated mother. To our knowledge, this is the first report on transmission of chemotherapy effects to several filial generations. The mechanisms for transgenerational transmission of the chemotherapy-related phenotypes remain to be determined. However, the observed chromosomal abnormalities and the increase in severity of symptoms in generations four and five, let us to hypothesize that DXR-induced damage in female gametes is characterized by chromosomal deletions that genetically compound over two generations [20], [21], [22], [23].

This manuscript highlights the importance and the need for long-term follow-up of survivors of cancer treatment and their offspring. Our data suggest that most of the childhood cancer survivors (approximately one in 900 adults in the United States) [24] –together with their offspring – might be at risk for late effects from chemotherapy. Results from the present experiments may provide valuable prognostic information for this growing human population; such data are pertinent considering that reproduction remains an option for adults in this population.

## Results

### DXR treatment resulted in permanent depletion of primordial follicles

**Figure 1:**
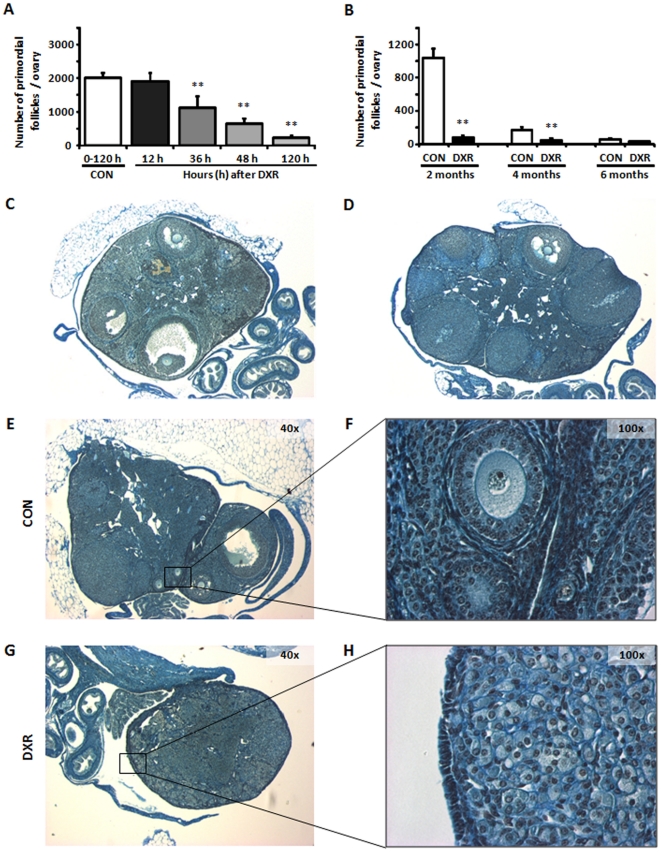
Assessment of ovarian reserve after *in vivo* exposure to DXR. (**A**,**B**) DXR caused depletion of the primordial follicle pool in a time dependent manner. ^**^ *P*<0.001 relative to CON; opened bars (vehicle CON); filled bars (DXR); the number of mice analyzed per group is provided in parentheses above each bar. (**C**,**D**) DXR did not affect follicles beyond those in the primary stage; as depicted in these micrographs, several antral follicles (black stars) and corpora lutea (white stars) are present in the ovaries of treated females. (**E**,**F**) Some primordial and small follicles were still present in the ovaries of CON females six months after treatment. (**G**,**H**) The ovaries of DXR-treated females at 6 months of age resembled the ovaries of very old females, with no follicular structures or corpus luteum present; moreover those ovaries had shrunk to approximately half size compared to CON ovaries from females of the same age, compare E and G.

Recently, it has been suggested that DXR-induced depletion of the primordial follicle pool is a temporary state, and that 48 h after a single intraperitoneal injection of DXR (5 mg/kg), replenishment of the primordial follicle pool somehow resumes and reaches control values by the second month after treatment [25]. In an effort to replicate such findings, we performed a series of experiments under the exact same conditions as Johnson et al. Our results confirmed those previously reported by us [26], [27], [28], [29] and others [29], [30], [31], [32] in that DXR causes depletion of the primordial follicle pool in a time dependent manner (Fig. 1A&B). On the other hand, DXR does not appear to affect follicles beyond those in the primary stage (Fig. 1C&D). The disappearance of primordial follicles was evident 36 h after a single intraperitoneal injection of DXR, and by two months their number was almost close to zero. Four and six months after the treatment, the primordial follicle pool remained almost totally depleted with less than 20 primordial follicles remaining in the ovaries (Fig. 1B, &E–H). Therefore, unlike Johnson et al., we did not observe replenishment of the primordial follicle pool; the reason for the discrepancy between our study and that of Johnson et al. [25] remains to be determined.

### DXR induced myometrial damage and death of treated mothers due to delivery complications

Contrary to the induction of damage and depletion of the primordial follicle pool, DXR does not appear to affect the pools of follicles already in the growing phase (Fig. 1C&D). Henceforth, we sought to determine what effects DXR has on the fertility of treated females. At seven weeks of age, female mice were given a single intraperitoneal dose of DXR (10 mg/Kg; N = 50). Two months after treatment, females were individually caged with young (8 weeks of age) adult males of proven fertility. As expected for a drug that does not affect the antral follicles and ovulation (Fig. 1C&D), there was no significant difference in pregnancy rates between control and the chemotherapy group. From 30 control females (receiving a single intraperitoineal injection of saline), 29 got pregnant and delivered within 30 days of being caged with males. These females all delivered normally, and the number of pups/litter was also normal (average 8±2 pups/litter). In the chemotherapy group (N = 50), 47 females got pregnant within 30 days of being caged with males. Unexpectedly, 66% of the DXR-treated females had dystocia and died during delivery. The remaining females (34%) delivered normally, although they had less pups/litter (average 4±1 pups/litter; p<0.05 compared to CON), which reflects the diminished primordial follicle pool in these females.

**Figure 2:**
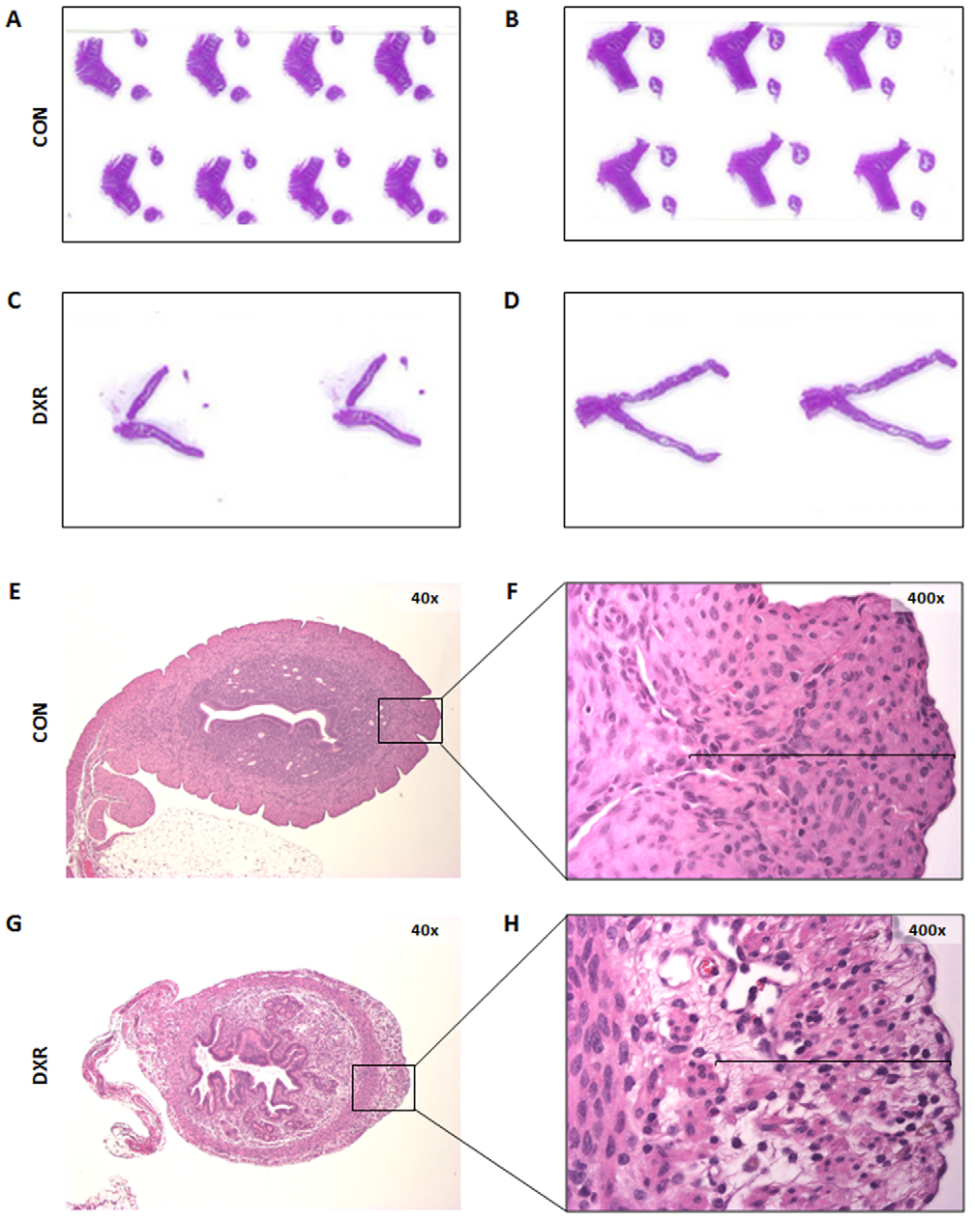
DXR induced atrophy of the myometrium. (**A–D**) Macroscopic appearance of uteri from two different females at two months after treatment with either saline (A,B) or DXR (C,D). Notice the difference in the thickness of the uterine horns. (**E–H**) Uterine sections stained with H&E. Notice the decrease in the thickness and sarcopenia of the outer myometrial layers of DXR-treated females (G,H) compared to control females (E,F).

Although, how DXR affects uterus function is unknown, previous studies found adverse side effects of DXR on various tissues (e.g. heart, brain, eyes, liver) [33], [34], [35], [36], [37]. Specifically, DXR decreases muscle mass and tone in some tissues [38], [39]. Therefore, we hypothesized that the delivery complications observed in DXR-treated females must have been the result of DXR actions on the uterus. We observed apparent macroscopic differences between control (Fig. 2A&B) and treated (Fig. 2C&D) females; uteri from treated females were smaller. Histological analysis of those uteri revealed that the thickness of the myometrial layers of DXR-treated females was highly decreased (5.7±0.25 mm, N = 30, Fig. 2G&H) compared to untreated females (10±1.3 mm, N = 30, Fig. 2E&F). In addition, in treated females, sarcopenia (decreased muscle fibers) of the outer myometrial layers was apparent (Fig. 2H), these side effects were observed in approximately 70% of treated females.

This finding could explain the delivery complications we observed. We conclude that the uterine anatomic changes contribute to the delivery complications observed in DXR-treated females. We postulate that the compromised uterine muscles in DXR-treated females could not generate enough force to complete delivery. Oxytocin injections (2 injections 30 min apart at the time of delivery) were not effective, in few DXR-treated females (2 out of 15 females) it only helped to deliver one pup, while the rest of the litter remained retained. The mothers died due to complications following dystocia, fetal maceration and bacterial infection. When C-section was performed at the first signs of parturition, the procedure was an efficient intervention to rescue the pups (N = 42 pups from 10 treated females) as opposed to oxytocin treatment.

### DXR affected both the oocyte and the uterine environment

**Figure 3:**
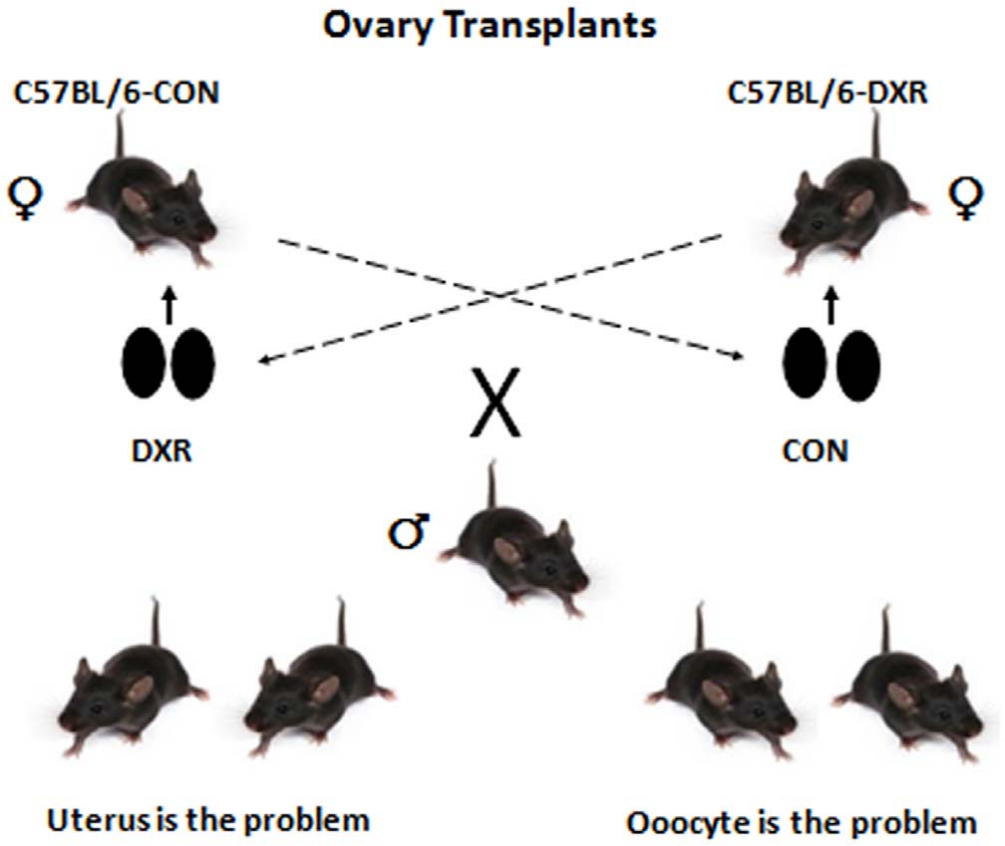
Schematic representation of the ovarian transplantation and mating trials. Control ovaries were transplanted into DXR-treated females, and DXR-treated ovaries were transplanted into CON females. Two weeks after surgery, all recipients were placed in mating trials with adult males of proven fertility.

We noticed that surviving pups born to a chemotherapy-treated mother were physically weaker compared to control. Therefore, to determine the role played by the oocyte and the uterine environment in the DXR-induced delivery complications and poor health of the neonates, we performed ovary transplants involving swaps between DXR-treated and control females (Fig. 3). Females were treated with a single intraperitoneal dose of either sterile saline (CON; N = 37) or DXR (N = 37) when they were 7 weeks of age. Six weeks post-treatment, the ovaries were surgically removed from both CON and DXR-treated females. The control ovaries were then transplanted into DXR-treated females (CONov-DXRuterus), whereas the DXR-treated ovaries were transplanted into CON females (DXRov-CONuterus). Two weeks after surgery, females were mated with young males of proven fertility. Five ovariectomized (OVX) control females that didn't receive ovaries were also caged with males, and as expected, no pregnancies were observed in any of them. Approximately 70% (26 out of 37) of control females receiving DXR ovaries (DXRov-CONut) got pregnant. However, 22% (6 out of 26) of those females had delivery complications and died; no histological alterations were observed in the myometrium of these females (data not shown). The rest of those females delivered normally, although the litters were smaller and the pups weaker; among those pups, the rate of neonatal death was 83% (from 36 pups born, 30 died during the first week). In the CONov-DXRut group, the pregnancy rates decreased to 35% (13 out of 37), and seventy percent of those pregnant females (9 out of 13) died at the time of delivery due to dystocia-associated complications. The remaining 30% (4 females) delivered a total of 8 pups, though smaller in size but otherwise normal.

These results suggest that pregnancy and delivery complications seen in DXR-treated females or females that got DXR-treated ovaries appear to be mostly linked to uterine health (78% contribution); however there was also a 22% contribution by the oocyte. Importantly, the health of the neonates appears to be primarily controlled by the oocyte (83% contribution). But, in those transplant experiments, the contribution of the immune system due to immunological rejection cannot be totally ruled out, even though the mice were syngeneic.

### DXR-induced effects on the oocyte were transmitted transgenerationally

**Figure 4:**
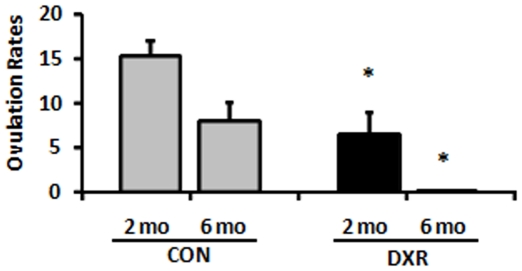
Ovulation rates of G0 females at two and six months after treatment. The ovulation rates of G0 females treated with DXR were markedly decreased compared to CON. ^*^ *P*<0.05; opened bars (vehicle CON); filled bars (DXR); the number of mice analyzed per group is provided in parentheses above each bar.

In an effort to elucidate the role that DXR-exposed oocytes play in the delivery complications, we monitored the offspring of the treated and control G0 females for six generations. Firstly, we tested if oocytes recovered from the G0-DXR females were more susceptible to DXR *in vitro*. Although, the ovulation rates of G0 females were markedly decreased (7±2.2 oocytes/female compared to 15±1.87 oocytes/female in the control; p<0.05; Fig. 4), those oocytes were equally sensitive to DXR as the control. By the time the G0-DXR females were 8 months of age no oocytes could be recovered after superovulation; we were unable to harvest immature oocytes from the ovary as well. In any case, no G0-DXR female delivered after 6 months of age, whereas control females kept delivering until approximately 10–12 months of age.

**Figure 5:**
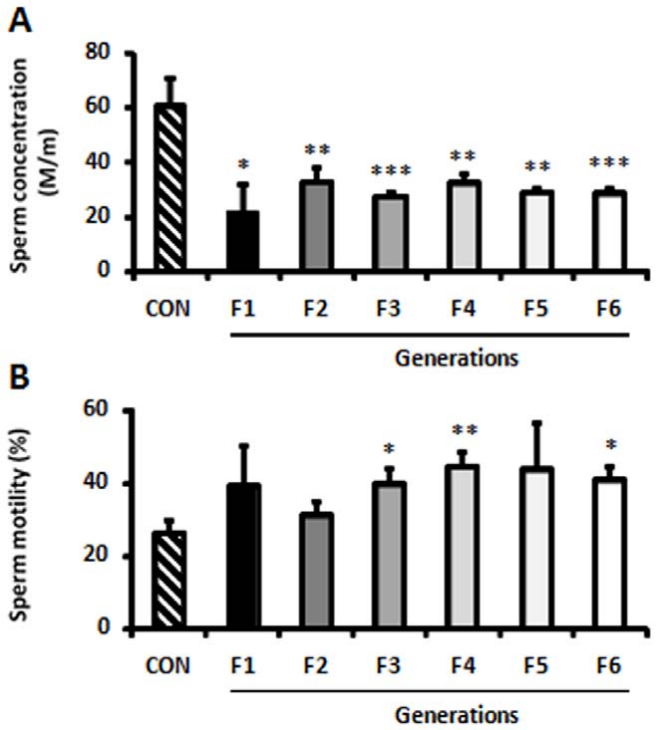
Sperm analysis for control males and males born to DXR-treated mothers over six generations. (**A**) A marked decrease in sperm concentration was observed at the first generation (G1) and remained below CON levels for the subsequent five generations studied. (**B**) Motility on the other hand appeared to improve over several generations. Control males of the same generations do not show significant changes in either the sperm concentrations or sperm motility compared to the Go. *P<0.05; **P<0.001; ***P<0.0001; opened bars (CON); filled bars (offspring of DXR-treated females); the number of mice analyzed per group is provided in parentheses above each bar.

**Figure 6:**
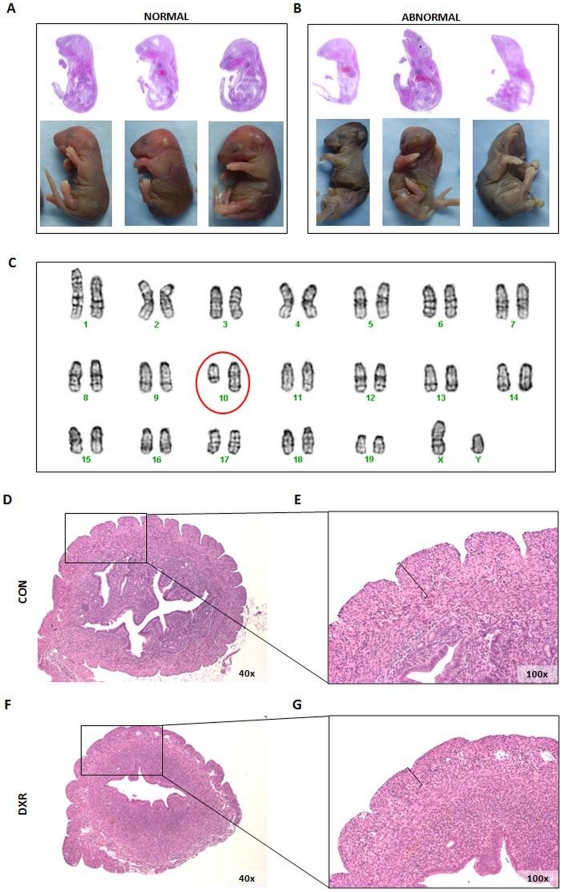
Representative images of transgenerational phenotypes observed in offspring of DXR-treated (G0) females. (**A**) Examples of normal pups (day 0) born to CON females. (**B**) Physical malformations, particularly head abnormalities were observed in G4 pups (day 0), but note that only the G0 female was treated with DXR. (**C**) Representative karyotype of abnormal G4 pups, depicting a large deletion on chromosome 10. (**D–G**) Histological appearance of uteri from control (D,E) and G4 (F,G) females. Notice the thinner myometria in G4 females although they were never treated with DXR; only the G0 females received DXR. Compare the marked layers in E&G. Note that none of these effects were seen in the control females of the same generations.

Pups that survived after the first week (from now on called G1) developed normally. Compared to CON, in G1-derived oocytes there were no significant differences in their ovulation rates or in the *in vitro* sensitivity of oocytes to DXR. Fertility rates of G1 males and G1 females were also not different from control (G1 N = 20 males and 20 females, and they produced approximately 130 pups here called G2). However, when semen analyses were performed, a marked decrease in sperm concentration was observed at the first generation (G1) and remained low for the subsequent six generations studied (Fig. 5). Note that control males of the same generations do not show significant changes in the sperm concentrations compared to the Go. In both sexes, there was a marked decrease in fertility in G2. In females, the most striking transgenerational effects were observed in G4 and G6, where we found increased rates of neonatal death and physical malformations, predominantly head abnormalities (Fig. 6A&B), chromosomal abnormalities, particularly deletions on chromosome 10 in all abnormal pups genotyped (N = 7; Fig. 6C), and death of mothers due to delivery complications (Table 1). The affected females exhibited thinner myometria (Fig. 6F&G) compared to females that delivered normally (Fig. 6D&E). Note that none of these effects were seen in the control females of the same generations.

**Table 1:** Reproductive outcomes by generation.

Generation	CON	G0	G1	G2	G3	G4	G5	G6
Number of females	50	50	30	30	30	37	30	30
AVG age at first pregnancy after treatment (mo)	4	4	2.5	2.5	2.5	2.5	2.5	2.5
AVG number of pregnancies over 8 mo breeding	7	1	6	3	4	4	7	7
AVG number of live pups over 8 mo breeding	9	3	8	5	5	3	8	6
Percentage of spontaneous early pregnancy losses	0	0	0	3	3	0	0	0
Percentage of females dying at delivery	0	56	0	0	6	9	0	0
Percentage of abnormal pups	0	0	0	0	1	7	0	0

### Positive correlation between deletions in chromosome 10 and the presence of ring-like cells

**Figure 7:**
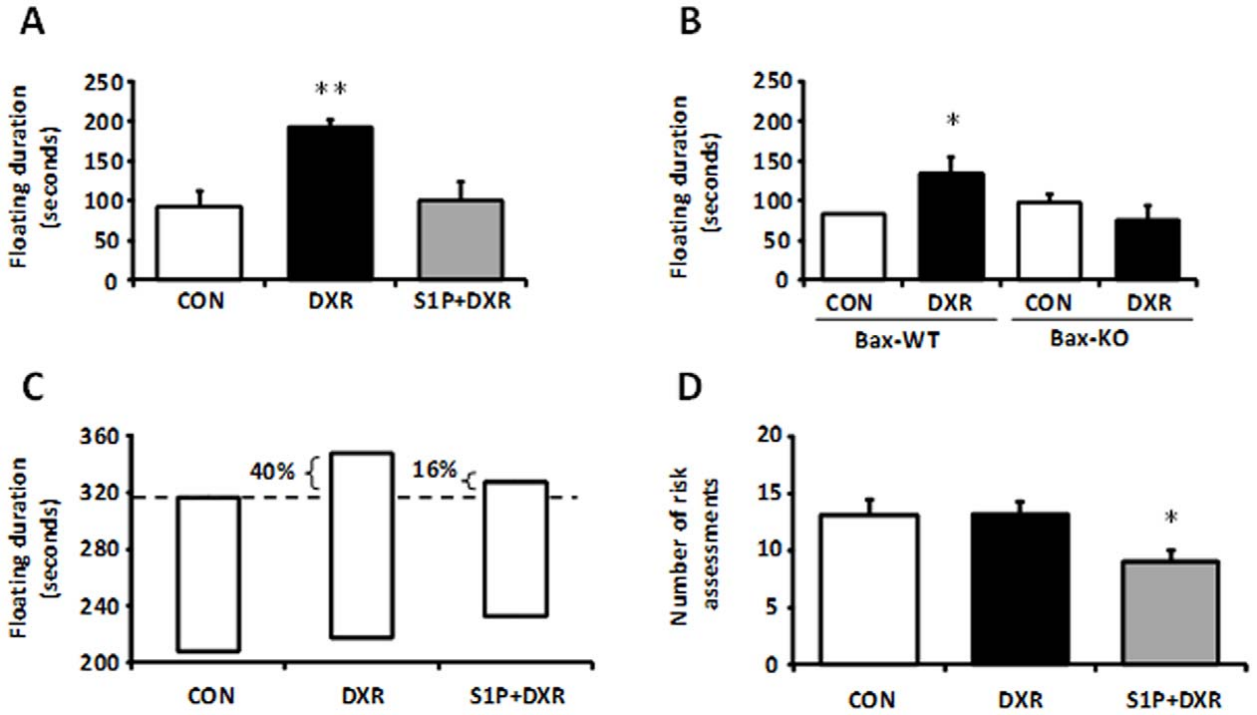
Neonatal death rate and percent of ring-like cells in peripheral blood of CON, G0 and their offspring over six generations. (**A**) Note the increased rates of neonatal deaths in G0, G4 and G6. (**B**,**C**) Significant numbers of ring cells were found in the peripheral blood of G3, and G5 parents (both males and females) that gave rise to either high rates of neonatal death and/or abnormal pups. (**D**) A positive correlation (r = 0.91) between the presence of ring-like cells in peripheral blood and the appearance of the abnormal pups in the following generation.

Following observation of high rates of neonatal death in G4 and G6 (Fig. 7A), and malformations in G4 (Table 1), we decided to karyotype those pups. Interestingly, the specimens from the pups exhibited a deletion in chromosome 10. Furthermore, during the karyotyping analysis, it was discovered that the cultures being karyotyped possessed rare cells called ‘ring- and/or hyposegmented-cells’. Intriguingly, we also found significant numbers of ring-like cells (Fig. 7D) in the peripheral blood of G3, and G5 parents (both males and females) that gave rise to either abnormal pups and/or pups that died during the first week of life (Fig. 7B&C). Moreover, we found a remarkably tight correlation (r = 0.91) between the presence of those cells in peripheral blood and the increase of abnormal pups and/or dead neonates in the following generation (Fig. 7A). Interestingly, a percentage of ring-like cells ≥2% in either parent directly correlates with problems seen in the generation immediately after.

### Short-term starvation (STS) decreased the duration of the initial distress caused by chemotherapy

Since DXR is a highly used and effective chemotherapeutic agent, it is important to identify approaches to either totally prevent or diminish its undesirable effects. Recently, it was proposed that fasting before chemotherapy could decrease the side effects in people undergoing chemotherapy [40], [41]. Herein, we found that in female mice, fasting for 48 h before a single dose of DXR decreases the early observable side effects of the drug, i.e., freezing behavior, ruffled hair, and hunched back posture. While DXR-treated females displayed those distressed signs for approximately 21 min (AVG 19±3 min; N = 30; DXR), in females undergoing STS before treatment this time span was shortened considerably (AVG 12±3 min; N = 30; STS+DXR), although still higher than control animals (immobility time average 0.4±0.2 min; N = 15). Fasting for 48 h also partially protected the myometrium (7.1±0.25 mm, N = 20; compared to DXR alone 5.7±0.25 mm, N = 30; p<0.05), but it didn't prevent delivery complications and death of mothers and pups. Apparently, losing 25% or more of the myometrial thickness could have devastating consequences. Fasting for 48 h appeared to partially protect the primordial follicle pool, although the difference was not significant (data not shown).

In humans the recommended fasting period is 72 h [41], so it remains to be determined if in mice increasing the fasting period by 24 h would have more dramatic benefits beyond those observed post 48 h STS. Since most complaints from chemotherapy patients refer to the immediate side effects of the drugs [36], we speculate, based on our results, that STS might indeed be useful in reducing such undesirable outcomes.

### DXR induced anxiety and despair-like behaviors that were corrected either by pre-treatment with S1P or deficiency of Bax

To continue our studies on the side effects of chemotherapy and how to either prevent or control them, we analyzed behavioral changes. We performed neurobehavioral phenotyping of the following groups of females: CON (N = 20); DXR (single dose, N = 25); and S1P+DXR (N = 30). Since, in earlier studies [27], [28], we reported that *bax*-KO mice are protected from many of the undesirable effects of DXR, in these experiments we included a fourth group consisting of *bax* knockout females (*bax*-KO, without and with DXR; N = 10 and N = 15, respectively).

We examined depression/despair-like behaviors by tail suspension test and forced swimming test [42]. In the ‘Tail Suspension Test’ [42] the animal is suspended by its tail from the ceiling of the chamber, the duration of immobility is then recorded and inferred as an index of behavioral despair. In this test, we found no significant differences between the groups.

**Figure 8:**
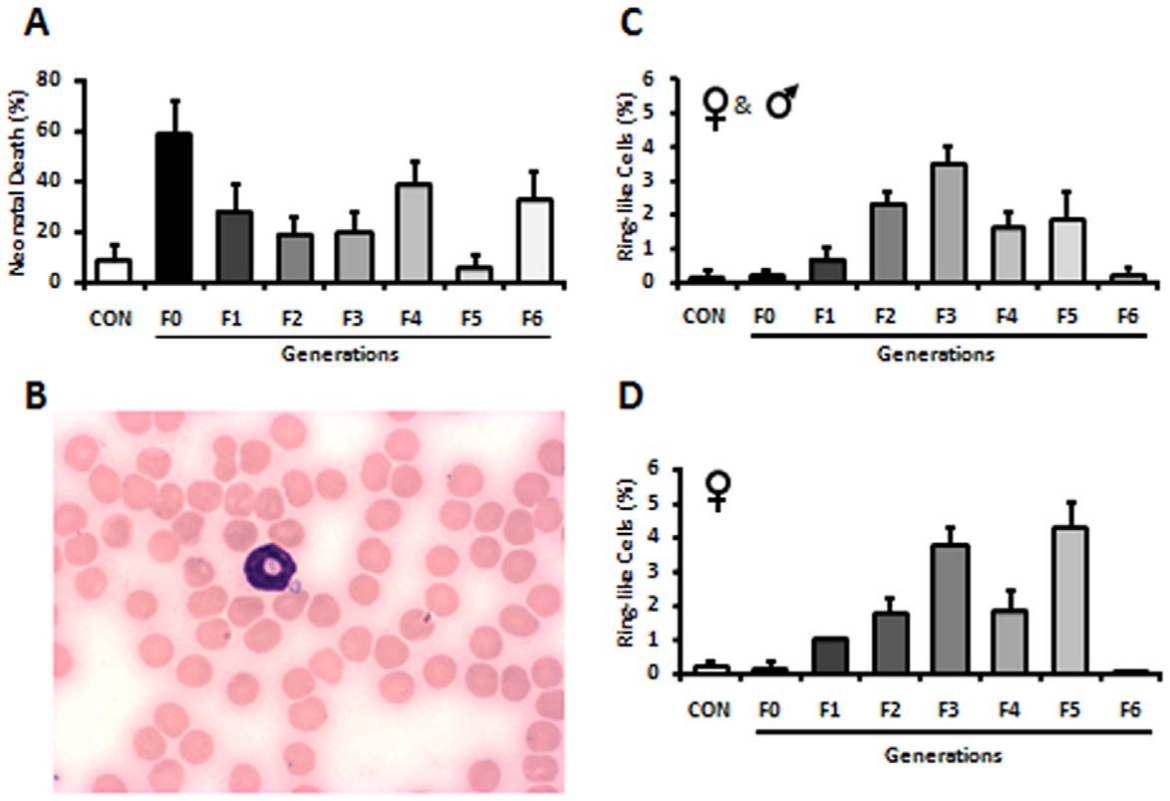
Behavioral phenotyping of G0 females. (**A–C**) The immobility observed in this test is considered to reflect a state of despair. CON, S1P+DXR, and *bax*-KO+DXR females floated less (i.e. remain less time immobile) compared to the DXR group, possibly indicating lower levels of despair in those females. While A&B illustrate the average time females remained floating, C depicts the percentage of females in the treated groups that remained floating longer than the control. (**C**) 40% of the females in the DXR group were above the maximum immobility time of the CON (i.e. more females in this group remain immobile and for longer periods); in the S1P+DXR group, only 16% of the females were over the maximum times in the control average. (**D**) Mice that perform more assessments and spend more time assessing the risk of moving out of the familiar environment are considered more anxious. Only the S1P+DXR group executed fewer risk assessments compared to the other two groups, indicating low level of anxiety. *P<0.05; **P<0.001; the number of mice analyzed per group is provided in parentheses above each bar.

The ‘Forced Swimming Test’ chronicles behavioral changes where mice are forced to swim in un-escapable situation. The immobility observed in this test is considered to reflect a state of despair. CON, S1P+DXR, and *bax*-KO+DXR females floated less (i.e. less immobility) compared to the DXR group (Fig. 8A–C), possibly indicating lower levels of despair.

As patients undergoing chemotherapy frequently complain about changes in behavior, particularly anxiety [34], [36], we evaluated anxiety levels in our mice using ‘Elevated Plus Maze’. In this test, when mice are initially introduced into a maze, they perform risk assessments before deciding to move onto any arm of the maze. More anxious mice spend more time assessing the risk (i.e. higher number of risks assessments) of moving out of the familiar environment (closed arms). Although we found no significant indication of anxiety in the DXR-treated females, it is worth mentioning that the S1P+DXR group differed significantly, since the females executed less risk assessments compared to the other two groups (Fig. 8D). This could be an indication of their lower level of anxiety. Therefore, it would be interesting to evaluate further the therapeutic potential of S1P as an anxiolytic drug. Unlike humans, female mice treated with chemotherapy seem to be more prone to despair and not anxiety.

## Discussion

The present report examines the effects of a single intraperitoneal injection of DXR on the reproductive and behavioral performance of adult female mice and their progeny. In earlier studies [13], [16], it was suggested that oocytes that survive DXR treatment might be less capable of being fertilized successfully. Our results demonstrate that this is not the case, as there was no difference in pregnancy rates between G0 treated and CON females. However, our studies unveiled important reproductive risks for both the treated mother and her G4 and G6 descendants (see below). The delivery complications we observed in G0 females treated with DXR have not been reported in humans. Nonetheless, it is important to note that human pregnancies following chemotherapy are considered high risk in nature and delivered via C-section [3], [8], [10], [11], [12], [43]; and as we reported here C-section was a very effective intervention to rescue pups and prevent delivery complications. An issue that we did not address in the present studies is whether or not G0 females that delivered normally would have had any problems in subsequent pregnancies.

In any case, no G0 female delivered after 6 months of age. This was expected since in these females the primordial follicle pool has been depleted (compare Fig. 1E to 1G) and hence the loss of reproductive capacity was about 4 months earlier in the treated G0 females than in control females. In women, this would be the equivalent of onset of menopause, and agrees with current knowledge on the induction of early menopause (occurring 8 years earlier) in women following chemotherapy treatment [2], [6], [7], [9].

That DXR is a female germ cell mutagen has been previously demonstrated [20], [44], [45], [46]. Those earlier studies [20], [44], [45] and the present one raise the possibility of transmissible genetic damage induced in germ cells. However, the chromosomal abnormalities observed in those studies and the ones reported here are different. This could be due to differences in either dose, strain of mice, and/or length of treatment. There are also earlier publications on the effects of chronically administered chemotherapeutic agents (e.g., cyclophosphamide) on the immediate offspring of mice and rats [47]. But, to our knowledge, there is no report on the transmission of any chemotherapy effects to several filial generations, and that's what makes the present manuscript unique. Therefore, retrospective and long-term prospective studies of the subset of human population are needed to ascertain the issues discussed. Our main motivation is to raise awareness about the importance and the need for long-term follow-up of survivors of cancer treatment and their offspring. Such data would be beneficial for genetic counseling of childhood cancer survivors; this subpopulation of approximately one in 900 adults in the United States [24] –together with their offspring– might be potentially at risk for the transgenerational effects from chemotherapy.

Although, with the present experiments we can not exclude the possibility as to whether the effects we saw were due to either DXR or any of its metabolites [19], [48]; it is clear that, a single dose of DXR was able to cause the observed plethora of undesirable effects in both the mother and her G4 and G6 descendants as well. As of date, 40 years after its first usage [49], transgenerational effects of DXR in the human population have not been reported. This might be due to the fact that the most devastating consequences we observed (i.e. increases in neonatal death rates and malformations), are expected to manifest at the earliest in G4. This means approximately 70 years after dosing the G0 parent, based on a hypothetical example of a girl (G0) getting treatment at the age of 10 years, delivering the G1 at the age of 21.4 (according to the CDC data from 1970; [50]), and assuming that the average age of first time moms for the subsequent generations increases at an average of 0.11 yearly.

As to how DXR induces all those effects, our data point to both genetic (chromosomal deletions) and non-genetic mechanisms as possibly underlying the different effects seen in the present studies. It has been proposed that DXR exerts antitumor activity by interacting with DNA and interfering with its metabolism [19]. However, some of the toxicity of this drug has also been related to interfering with mitochondrial functions [51], [52]. In general, enzyme inhibitions [19], lipid peroxidations [53], membrane disorders and oxidative stress [34], [51], are now being associated with the toxic side effects of DXR. Although at this time we cannot pinpoint the exact mechanism(s) involved in the transgenerational effects, several facts from our results led us to speculate that they can be partly attributed to dysfunction of mitochondria and inheritance of those dysfunctional organelles. Our first conviction is based on observation of acceleration of aging-like phenotypes in ovary, uterus, and brain; each one of those tissues exhibited degenerative changes previously attributed to mitochondrial dysfunction [34], [54], [55]. Secondly, there is the fact that, the phenotype skips generations, which reflects a landmark of mitochondrial inheritance [56], [57], [58], [59], [60]. And thirdly, more individuals in a litter are affected when the mother (and not the father) is the suspected carrier. Note that practically all mitochondria in an individual are provided by the oocyte [58], [61], [62]. Furthermore, considering the fact that mitochondria are crucial for maintaining oocyte health and developmental potential [34], [54], [62], and since oocytes are the cells in the body with the higher number of mitochondria [58], [62], one can envision why these organelles could be the most likely targets of DXR-induced damages in oocytes of the G0 female.

The observed chromosomal abnormalities and the increase in severity of symptoms in generations four and six, led us to hypothesize that DXR-induced damage in female gametes is characterized by chromosomal deletions that genetically compound over two generations [20], [44], [45]. DXR shows a wide range of effects on mammalian oocytes [7], [20], [23], [26], [44], [45], therefore, additional analysis is needed for a global view of oocyte response to this agent at the genome level [63]. Recent efforts in our laboratory are oriented towards oocyte genomic profiling and identification of the genes present in the deleted region of chromosome 10. Such analysis would yield insight into the molecular mechanisms of DXR injury in oocytes and other tissues as well, and would help in the rationale of designing more effective treatment strategies to circumvent the unwanted side effects of this drug.

Based on the percentages of ring-like cells in peripheral blood of offspring of the G0 treated female, we were able to predict male and female carriers (possess higher risk of producing abnormal litters) with an accuracy of 90% or higher. However, the exact relationship between the ring-like cells and the chromosomal aberrations observed is not yet clear in terms of etiology. Nonetheless, this correlation between a cellular defect and a phenotype is marked for our future genetic studies, and may also provide a potential biomarker for a diagnostic/screening tool in human clinics.

Neutrophils with ring-shaped nuclei have been described in human patients with myeloproliferative hematologic diseases [64], [65]. In our studies the mice possessing the ring-like cells look normal, and except for females that died due to delivery complications, we did not observe any other complications. Hence, most probably we can exclude the presence of a myeloproliferative disease. However, we cannot totally rule out the possibility that the mice could develop the disease later in life, most probably beyond 15 months of age; unfortunately in these studies we never kept our mice beyond this age.

Thus, we have shown that a reduction in the fertility of female mice and their offspring after DXR treatment can occur and can be attributed to genetic alterations or cell-killing events in oocytes or, presumably, toxicosis in non-ovarian tissues. DXR effects are not germ cell specific, and therefore the potential for failure of multiple biochemical pathways and deregulation of gene expression should be considered when trying to understand cell damage due to DXR.

## Materials and Methods

### Animals

For all experiments C57BL/6 mice were purchased from Charles River Laboratories at 6 weeks of age. Mice were kept in well-controlled animal housing facilities. Experiments were performed when the females were 7-week old. Their access to water and food was unrestricted, except in the experiments under short time starvation when food was removed for 48 hours. In the first set of experiments in which we replicated the studies by Johnson et al. [25], DXR was given as a single intraperitoneal dose of 5 mg/Kg. In all the other experiments, females G0 were treated with either a single intraperitoneal injection of DXR (10 mg/Kg) [26]; or saline, or two doses of S1P (Biomol, Plymouth Meeting, PA, USA; 20 µg/dose diluted in 50 µl) [66], [67]; via retroorbital injections at 18 h and at 30 min prior to the DXR treatment. All experiments involving animals described herein were reviewed and approved by the institutional animal care and use committee (IACUC) of Michigan State University (IACUC 12-07-178-00).

### Histomorphometric analysis of follicle numbers

Ovaries were collected at 12 h, 36 h, 48 h, 120 h, and at 2, 4 and 6 months after DXR treatment. Control ovaries were collected from vehicle-treated animals. Tissues were fixed (0.34 N glacial acetic acid, 10% formalin and 28% ethanol), embedded in paraffin and serially sectioned (thickness, 8 µm). The serial sections from each ovary were stained with hematoxylin and picric acid methyl blue, and every tenth section was analyzed for the number of healthy (non-atretic) primordial, primary and small preantral follicles, as described [26], [28], [68], [69]. For animals six months and older, every single section was subjected to counting (to avoid missing any primordial follicle).

### Histomorphometric analysis of myometrial thickness

Uteri were collected at 12 h, 36 h, 48 h, 120 h, and at 2, 4 and 6 months after both DXR treatment and control. Tissues were fixed, embedded in paraffin and sectioned (thickness, 8 µm). For each female, two or three full cross- and longitudinal-sections of uteri were stained with hematoxylin and eosin, photographed, and myometrial thickness measured in the largest full cross-section.

### Ovarian transplantation and mating trials

As described [70], six weeks after the DXR treatment, one control and one DXR-treated female were anesthetized, and prepared for aseptic surgery. Both ovaries of each female were removed through a single dorsal skin incision across the lumbar area, and placed in separate (CON or DXR) Petri dishes containing 2 ml of modified human tubal fluid (Irvine Scientific, Santa Ana, CA). Next, CON ovaries (left and right) were transplanted into the corresponding empty ovarian bursas of the DXR-treated female. The incision was closed with surgical clips. The same procedure was then repeated for transplanting the DXR ovaries into CON females. Two weeks after surgery, all recipients were placed in mating trials with adult males of proven fertility. The mating trials continued until the females were 15 months of age, or until death, which usually occurred prior to 15 months in the treated animals.

### Oocyte Collection and Culture

Female mice were superovulated with a hormonal regimen of eCG/hCG as previously described [26], [54], [71]. Mature oocytes were collected from the oviducts 16 h after hCG injection and cultured *in vitro* as previously described [26], [54], [71]. Assignment to treatment groups was carried out at random, and incubations for up to 24 h performed without or with freshly made DXR (200 nM; Alexis Biochemicals, San Diego, CA).

### Analysis of Apoptosis

At the end of the culture period, the oocytes were fixed and evaluated as detailed previously [27], [72], [73], for characteristics of apoptosis, including morphological changes (*e.g*., condensation, budding and cellular fragmentation) and biochemical alterations (*i.e*., DNA cleavage; Comet Assay Kit, Trevigen, Gaithersburg, MD). The percentage of oocytes that underwent apoptosis out of the total number of oocytes cultured per drop in each experiment was then calculated.

### Semen analysis

Sperm were collected from cauda epididymis and capacitated *in vitro* for 1 h. Immediately after, sperm motility (path velocity, progressive velocity and track speed) and concentration were evaluated by computer-assisted sperm analysis (CASA) using Hamilton-Thorne IVOS 12.3 as previously described [74].

### Behavioral phenotyping

Behavioral analyses for anxiety (elevated plus maze), despair and depression (tail suspension and forced swimming tests) were performed at the Toronto Center for Phenogenomics (www.cmhd.ca) using standard approved procedures [75].

### Karyotyping

C-banded karyotypes of somatic chromosomes from live cultures and tissues were performed by Cell Line Genetics (Madison, WI), using standard protocols [76].

### Blood smears

A small drop of blood was collected from the tail; blood smears were prepared, allowed to dry, fixed with methanol, and stained with Wright Giemsa staining. All slides were read by an expert pathologist having no knowledge of the experimental groups.

### Data presentation and statistical analysis

All experiments were independently repeated for at least three times (unless stated otherwise) with different sets of mice. Data not differing significantly were pooled by groups. The combined data from the replicate experiments were subjected to a one-way analysis of variance followed by Scheffe's F-test, Fisher's exact test or Student's *t*-test. Differences between group means were considered statistically significant at *P*<0.05. The data depicted in graphs represent the mean ± SEM of the combined data.
