# Correction: Chemotherapy-Induced Late Transgenerational Effects in Mice

**DOI:** 10.1371/annotation/4fffb4ef-31ad-4aab-bfce-2c97522c9af4

**Published:** 2011-03-24

**Authors:** Loro L. Kujjo, Eun A. Chang, Ricardo J. G. Pereira, Shilpa Dhar, Brenda Marrero-Rosado, Satyaki Sengupta, Hongbing Wang, Jose B. Cibelli, Gloria I. Perez

The legends for Figure 7 and Figure 8 were switched in production; the legend for Figure 7 should be that of Figure 8 and vice versa. The figures themselves are correct.

